# Stress as a possible cause of a high incidence of hypertension and diabetes and a low incidence of asthma in the Iraqi population

**DOI:** 10.25122/jml-2022-0266

**Published:** 2023-03

**Authors:** Zahraa Al-Isawi, Salim Kadhim, Yahya Yahya, Najah Rayish Hadi

**Affiliations:** 1 Department of Pharmacology and Toxicology, Faculty of Pharmacy, University of Kufa, Kufa, Iraq; 2 College of Pharmacy, University of Alkafeel, Kufa, Iraq; 3 Department of Cardiovascular Sciences, University of Leicester, Leicester, UK; 4 Department of Pharmacology and Therapeutics, Faculty of Medicine, University of Kufa, Kufa, Iraq

**Keywords:** hypertension, diabetes, asthma, stress

## Abstract

Hypertension and diabetes represent a significant public health burden worldwide and are significant risk factors for heart disease and stroke. Nevertheless, Iraqi people, in particular, experience higher levels of stress due to political instability and economic issues. The study aimed to investigate the prevalence of common morbidities among Iraqi patients and the possible relationship with exposure to stress. The data was collected from patients (n=500) who attended the health center in Najaf, Iraq, between 25 August 2021 and 30 September 2021. The prevalence of hypertension, diabetes, and asthma among Iraqi people was determined along with patients’ awareness and control of these conditions. In addition, patients were asked about their experiences with stress, including the type of stress they encountered. Our findings revealed that nearly 47% of patients involved in this study had hypertension, with the highest percentage in patients over 40. Moreover, the incidence of diabetes was 12%, with the highest incidence in the age group of 40-59. The incidence of asthma was lower in all groups. Data analysis concerning exposure to stress demonstrated that about 60% of patients suffer from a stressful life. We found that the incidence of hypertension and diabetes was high while the incidence of asthma was low. This study also reveals that a considerable number of people were unaware of their hypertension and diabetes. Exposure to daily life stress among Iraqi people may play a role in the observed incidence of these morbidities.

## INTRODUCTION

Hypertension is one of the most common non-communicable diseases worldwide, responsible for a significant number of disabilities and deaths [1]. It is a main risk factor for heart disease and stroke (average blood pressure [BP] reading of 140/90 mm Hg or greater), affecting about 20% of the world's population (over a billion people). The global prevalence of hypertension has been rising and is expected to continue to rise in the next decade, despite decreases in elevated blood pressure (BP), mainly in high-income countries, which can be partly attributed to improved detection and treatment [2]. In 2015, hypertension was the cause of 10.7 million deaths and 211.8 million disability-adjusted life years around the world. As the incidence of diabetes in young and middle-aged adults rises, so does the interest in determining the role of stress in a person's long-term survival following the onset of diabetes. However, we only found a few studies examining how long-term exposure to stress or noise affects the survival of people with diabetes. People worldwide are affected by bronchial asthma and chronic obstructive pulmonary disease (COPD), both debilitating lung diseases. In particular, children are affected by asthma, which is a public health issue. There are currently 235 million people with asthma, according to the World Health Organization (WHO) estimate, but this figure is expected to increase because asthma is underdiagnosed and under-treated. The causes of asthma are not fully understood, but environmental factors, such as house dust mites, cockroaches, carpet pollens, active and passive tobacco smoke as well as air pollution, may cause the airways to become inflamed due to genetic predisposition and environmental exposure [3,4]. Stress is a part of daily life and can affect individuals in various ways. While being stressed at some point in life is common for everyone, there is an interindividual variation in how people deal with stress. [5]. Stress is characterized by feelings of frustration, anger, and nervousness [6]. While many individuals can manage stress well, it may be accompanied by physical and/or psychological symptoms [7]. To a certain extent, stress can be beneficial, helping an individual to deal with situations and perform tasks. Nevertheless, it has been shown that excessive stress can cause or worsen various medical conditions. Stress may be characterized by physical symptoms such as fatigue, headache, dizziness, gastrointestinal (GI) upset, muscle tension, weight loss (or gain), and back pain or psychological symptoms such as irritability, anger, depression, nervousness, and anxiety. In addition, stress may lead to sleep disturbances [8]. The American Psychological Association (APA) categorizes stress into acute and chronic [9]. Modern lifestyle factors like insufficient physical activity, a poor diet (particularly high salt intake), noise, and alcohol consumption are major contributors to hypertension [10]. According to the World Health Organization, “diabetes is a chronic disease that occurs either when the pancreas does not produce enough insulin or when the body cannot effectively use the insulin it produces” [11]. Insulin is a hormone that regulates blood sugar. Diabetes has nearly doubled in prevalence between 1980 and 2014, affecting approximately 422 million adults worldwide [11]. Since the study of behavioral risk factors like smoking and physical activity has taken place in several countries, environmental risk factors have received far less attention from intervention efforts. The number of studies investigating the link between cardiovascular diseases and environmental factors, such as stress, is growing. According to the American Heart Association recommendation, more research must be conducted to examine the role of stress [12]. Statistical surveillance performed by the APA in 2007 showed that more than 33% of Americans suffer from high-stress levels. In addition, more than 20% of individuals suffer from high-stress levels 15 days or more each month. Money, work, and housing crisis are among the major causes of stress [13].

This study was designed to examine the prevalence of hypertension, diabetes, and asthma among Iraqi people and their association with age, awareness, and control. Furthermore, the study aimed to explore the relationship between these morbidities and the level of stress experienced by the patients in their daily lives. By addressing these relationships, the study can expand our current understanding of the incidence of these diseases in Iraq and highlight the importance of reconsidering age-related incidence and standard measurements of diseases under investigation. Lastly, the study underscores the importance of increasing awareness and knowledge about the risk factors and management of these chronic diseases in improving the prognosis and outcomes of affected individuals.

## MATERIAL AND METHODS

### Participants

This study included a total of 500 male patients who met the inclusion criteria, which covered the presence of comorbidities such as hypertension, diabetes, and asthma, as well as specific age ranges (18-39, 40-59, and over 60 years). It is important to note that the data was collected by male staff in a facility designed to receive male patients only; hence, female patients were excluded to avoid potential confounding factors such as hormonal changes and pregnancy. Additionally, patients below the age of 18 were also excluded from the study.

### Identification of diseases

Patients with hypertension, diabetes, and asthma were identified, as mentioned in Table 1.

**Table 1:** Identification of diseases.

**Identification criteria for patients with hypertension**	**Percent**
Seeking antihypertensive drug repetition	30%
Seeking blood pressure monitoring after antihypertensive medication	20%
Mentioned hypertension when asked before giving another treatment	50%
**Identification criteria for patients with diabetes**	**Percent**
Seeking antidiabetic drugs repetition	20%
Seeking blood glucose monitoring after antidiabetic medication	50%
Mentioned diabetes when asked before giving another treatment	30%
**Identification criteria for patients with asthma**	**Percent**
Seeking bronchodilator/steroid repetition	40%
Mentioned asthma when asked before giving NSAIDs	60%

### Place and time of data collection

The data for this study was collected from a health service station affiliated with the University of Alkafeel, located in Najaf, between August 25th and September 30th, 2021.

### Incidence rate calculation

The incidence rates of hypertension, diabetes, and asthma were calculated using several methods.

Incidence as percent = Number of cases for each age group/Total number of population*100%

Crude incidence rate = Number of cases for each age group/Total number of population*100,000

The age-adjusted incidence rate = Age distribution of standard population*crude rate

Age distribution of standard population = the number of participants for each age group/Total standard number of populations. For the Iraqi population, the standard number of population and age groups in 2021 was obtained from the central statistical organization/Iraq [14].

### Data analysis and presentation

Both data analysis and presentation were performed using GraphPad Prism software (version 9.3.1), USA. Data are presented as a percent or as the number of participants/patients.

## RESULTS

### Participants characteristics

The study participants (n=500) were categorized into different age groups, as presented in Table 2. The results showed that 34.4% of the participants were between 18 and 39 years, 36% were between 40 and 59 years, and the remaining 29.6% were above 60 years old.

**Table 2:** Classification of participants according to age group.

Age group	Number of participants	%
18–39	172	34.4
40–59	180	36
>60	148	29.6

### Demographic characteristics of participants

Table 3 shows the demographic data of the participants in terms of their residency and marital status. Most participants lived in urban areas, with 280 out of the 500 participants (56%) residing in urban locations and 44% residing in rural areas. Additionally, most participants (69.2%) were married, while 30.8% were not.

**Table 3:** Demographic characteristics of participants.

Feature	Age group			No. of participants	%
	18–39	40–59	>60		
**Residency**					
Urban	92	98	90	280	56
Rural	80	82	58	220	44
**Marital status**					
Married	70	134	142	346	69.2
Single	102	46	6	154	30.8

### Incidence of common morbidities among the Iraqi population

Table 4 presents the incidence of hypertension, diabetes, and asthma among the different age groiups. The results indicate that hypertension was more prevalent among older age groups, with an incidence of 24%, 60%, and 60.1% in the age groups of 18-39, 40-59, and over 60, respectively. On the other hand, the incidence of diabetes was lower among younger age groups, with a rate of 8.9% in the age group of 18-39, and higher in the older age groups, with rates of 15% and 14.8% in the age groups of 40-59 and over 60, respectively. Additionally, the incidence of asthma was relatively low across all age groups, with rates of 3.4%, 2.9%, and 3.4% in the age groups of 18-39, 40-59, and over 60, respectively, as shown in Table 4.

**Table 4:** The incidence of hypertension, diabetes, and asthma among the Iraqi population.

Disease	Incidence Percent according to age group		
	18–39	40–59	>60
**Hypertension**	24%	60%	60.1%
**Diabetes**	8.9%	15%	14.8%
**Asthma**	3.4%	2.9%	3.4%

### The age-adjusted incidence of hypertension

The crude and age-adjusted incidence of hypertension in the adult Iraqi population is shown in Table 5. The age-adjusted incidence is still high when compared to other countries. As shown in Table 5, the age-adjusted incidence of hypertension in the adult Iraqi population was 19465, 17422, and 6216 for age groups 18-39, 40-59, and more than 60 years, respectively. Such incidence is high when compared to other countries (see discussion).

**Table 5:** Age-adjusted incidence of hypertension in the adult Iraqi population.

Age group	Count	n	Crude incidence	Standard Iraqi population (2021)	Age distribution of standard population	Age-adjusted incidence
**18–39**	54	172	31395	4821313	0.62	19465
**40–59**	112	180	62222	2186306	0.28	17422
**>60**	92	148	62162	666699	0.1	6216
**All age groups**	-	-	-	7674318	1	43103

Count – the number of cases; n – the size of sample.

### The age-adjusted incidence of diabetes

Table 6 presents the crude and age-adjusted incidence of diabetes in the adult Iraqi population. The results indicate that the age-adjusted incidence of diabetes was 5572, 4200, and 1486 for age groups 18-39, 40-59, and above 60 years, respectively. These findings suggest a high age-adjusted incidence of diabetes in Iraq compared to other countries (see discussion).

**Table 6:** Age-adjusted incidence of diabetes in the adult Iraqi population.

Age group	Count	n	Crude incidence	Standard Iraqi population (2021)	Age distribution of standard population	Age-adjusted incidence
**18–39**	16	172	8988	4821313	0.62	5572
**40–59**	27	180	15000	2186306	0.28	4200
**>60**	22	148	14860	666699	0.1	1486
**All age groups**	-	-	-	7674318	1	11258

Count – the number of cases; n – the size of sample.

### The age-adjusted incidence of asthma

Table 7 displays the crude and age-adjusted incidence of asthma in the adult Iraqi population. The results indicate that the crude incidence of asthma was 2522, 1242, and 407 for age groups 18-39, 40-59, and above 60 years, respectively. These findings suggest a relatively low incidence of asthma in Iraq compared to other countries.

**Table 7:** Age-adjusted incidence of asthma in the adult Iraqi population.

Age group	Count	n	Crude incidence	Standard Iraqi population (2021)	Age distribution of standard population	Age-adjusted incidence
**18–39**	6	172	3488	4821313	0.62	2522
**40–59**	5	180	2900	2186306	0.28	1242
**>60**	6	148	3488	666699	0.1	407
**All age groups**	-	-	-	7674318	1	4171

Count – the number of cases; n – the size of sample.

### The incidence of hypertension, diabetes, and asthma in the adult Iraqi population (over 18 years)

**Figure 1:**
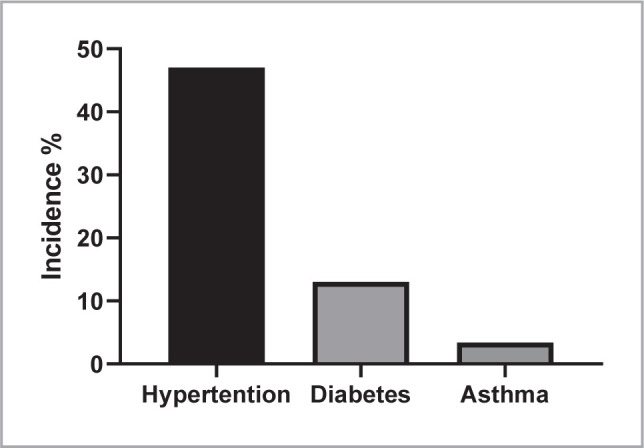
The incidence of hypertension, diabetes, and asthma in the adult Iraqi population (as percent).

Data depicted in Figure 1 highlight the incidence rates of hypertension, diabetes, and asthma among the adult Iraqi population aged 18 and above. Our findings indicate a high incidence of hypertension at 47% and diabetes at 13%, whereas asthma incidence was relatively low at 3.4%.

### Awareness and control of hypertension and diabetes within each age group

Table 8 shows the classification of participants according to awareness and control of hypertension within each age group. The majority of participants were unaware of their hypertension status, and there were a significant number of participants with uncontrolled hypertension. The percentage of controlled hypertension increased with age.

**Table 8:** Classification of participants according to awareness and control of hypertension (as percent).

Age group	Number of participants			
	Unaware	Controlled		Uncontrolled
		Non-pharmacological	Antihypertensive	
**18–39**	24.4	12.2	60	2.6
**40–59**	22.2	13.8	52.7	11.3
**>60**	5.6	16.8	66	11.6

A considerable number of participants were unaware of their diabetes, with the highest percentage in the age group 18-39 (18%) (Table 9). Most participants in all age groups managed their diabetes with antidiabetic medication, with the highest percentage in the age group over 60 (61%). However, a significant number of participants in all age groups did not control their diabetes, with the highest percentage being over 60 (25%).

**Table 9:** Classification of participants according to awareness and control of diabetes (as percent).

Age group	Number of participants			
	Unaware	Controlled		Uncontrolled
		Non-pharmacological	Antihypertensive	
**18–39**	18	22	50	10
**40–59**	12	15	53	20
**>60**	4	10	61	25

### Awareness and control of hypertension and diabetes in the adult Iraqi population (over 18)

The study revealed that a significant proportion of the adult Iraqi population above the age of 18 was unaware of their hypertension and diabetes conditions. Approximately 16.3% of participants were unaware of their hypertension, while 10.8% were unaware of their diabetes condition. Most of those aware of their hypertension and diabetes conditions achieved control, with 59% of hypertensive patients controlled with antihypertensive medications and 55% of diabetic patients controlled with antidiabetic medications. Additionally, non-pharmacological therapies were effective in controlling both hypertension and diabetes in a significant proportion of patients, with 14.6% of hypertensive patients and 15% of diabetic patients achieving control through non-pharmacological means. However, there was a significant proportion of patients with uncontrolled conditions, with 10% of hypertensive patients and 19% of diabetic patients experiencing an uncontrolled disease (Table 10).

**Table 10:** Awareness and control of hypertension and diabetes in the Iraqi population (as percent).

Age group	Number of participants			
	Unaware	Controlled		Uncontrolled
		Non-pharmacological	Treatment	
**Hypertension**	16.3	14.6	59	10
**Diabetes**	10.8	15	55	19

### Comorbidity

Table 11 shows the co-occurrence of hypertension, diabetes, and asthma in the studied population. The highest incidence of comorbidity was observed in the age group over 60, where 60 participants had both hypertension and diabetes. In the age group of 40-59, 25 participants had both diabetes and asthma, while in the same age group, 15 participants had hypertension and asthma. In the age group of 18-39, 10 participants had both hypertension and diabetes, while 7 participants had hypertension and asthma. The lowest incidence of comorbidity was observed in the age group of 18-39, where only 5 participants had all three conditions.

**Table 11:** Comorbidity of hypertension, diabetes, and asthma (as numbers).

Age group	HT/DM	HT/asthma	DM/asthma	HT/DM/asthma
**18-39**	10	7	10	5
**40-59**	40	15	25	3
**>60**	60	15	15	2

### Exposure to stress

**Figure 2:**
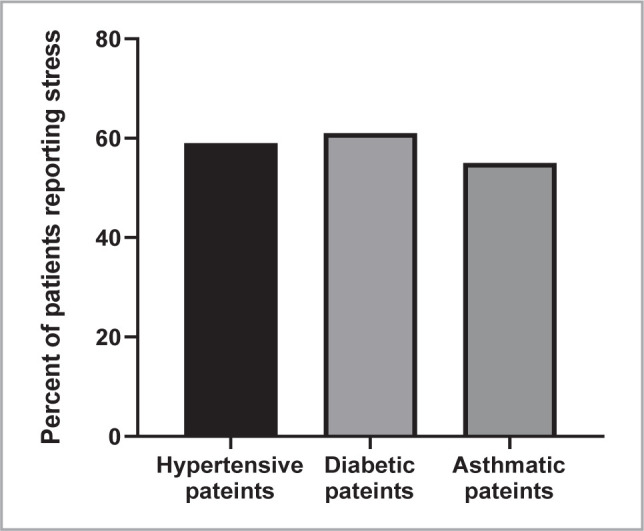
The percent of people under stress in each disease group.

About 58% of the participants reported suffering from stress. Among them, 59% were hypertensive, 61% were diabetics, and 55% were asthmatic, as shown in Figure 2.

### Causes of stress

**Figure 3:**
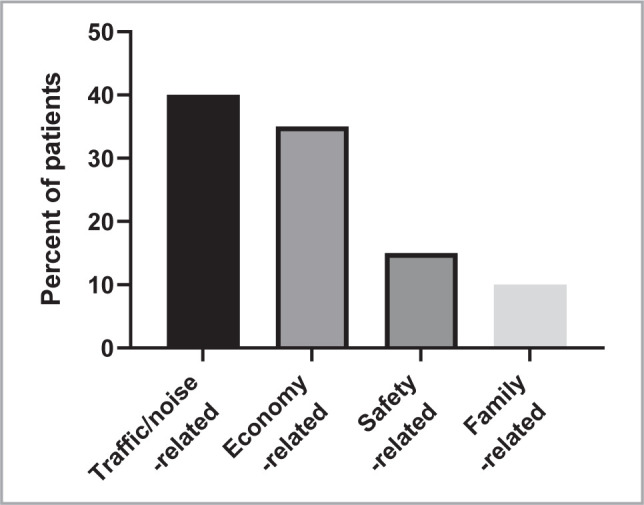
The common causes of stress in the adult Iraqi population (as percent).

Figure 3 presents the causes of stress reported by the participants. Traffic or noise was identified as a source of stress by 40% of the participants, while economic issues were reported by 35% of the participants. Safety concerns were mentioned by 15% of the participants, and family-related issues were the cause of stress for 10% of the participants.

## DISCUSSION

Our findings revealed that nearly 47% of the adult population involved in this study had hypertension, with the highest percentage in patients aged 40 years and above. Nevertheless, the incidence was high in the age group 18 to 39 compared to other countries. In addition, the incidence of diabetes in the adult population in this study was 12%, with the highest incidence in the age group 40-59. Moreover, the prevalence of diabetes in the young age group (18-39) was higher than in other countries. On the other hand, a lower incidence of asthma was shown in all age groups. Overall, the study evaluated the incidence of hypertension, diabetes, and asthma in the Iraqi population and showed a relatively higher incidence of hypertension and diabetes, along with a lower incidence of asthma, compared to several countries. The study compared the incidence of these diseases in Iraq with that of the United Kingdom [15-18], the United States [19,20], Saudi Arabia [21-23], and Egypt [24-26]. Each country was selected based on a specific criterion to enable meaningful comparisons. The UK and USA have highly rated medical services, medical research, and well-documented databases about diseases and their incidence. Although the economic status, safety, and public services are better in the KSA, people in both countries share several similar behaviors, unhealthy diets, sedentary lifestyles, and similar weather. Egypt was chosen as a comparison country due to its high population density, poverty, unsatisfactory economic status, and physically demanding jobs, comparable to conditions in Iraq. Compared to that in the UK, USA, KSA, and Egypt, the incidence of hypertension and diabetes in the Iraqi population is higher in all age groups. However, there are several interesting observations that need to be emphasized.

First, the incidence of hypertension and diabetes in the Iraqi population is relatively high and is particularly noticeable among younger individuals. Second, the incidence rates of hypertension and diabetes in Iraq are close to those in Egypt, which may indicate that stress is a considerable factor in the development of hypertension and diabetes. Several factors could be contributing to the high prevalence of hypertension among Iraqis, and one possible explanation is that stress may be a primary factor. Stress can cause a rise in blood pressure for short periods, but it can also lead to long-term high blood pressure. A number of the bodily systems that regulate arterial pressure are likely to be the same systems that have been suggested to explain the contribution of environmental stress to physical illness [27-28]. Neuroendocrine and autonomic nervous systems, as well as various brain regions, such as the hypothalamus, brain stem, and limbic system, all play a role in these processes. It is reasonable to assume that stress and high blood pressure are linked because the physiological mechanisms are closely linked.

People in their 60s and 70s are being diagnosed with diabetes at an alarming rate. In 2017, there were 123 million people over 65 with diabetes in the world, and that number is expected to double by 2045. Frailty, cognitive impairment and dementia, urinary incontinence, traumatic falls and fractures, disability, and the side effects of polypharmacy are all common geriatric syndromes that can have a significant impact on a patient's quality of life and interfere with anti-diabetic treatment in older patients with diabetes. Managing type 2 diabetes in elderly patients is a real challenge for the physician because of these factors [29]. Asthmatics and non-asthmatics in various cross-sectional studies have had their cortisol levels examined, with varying degrees of success. Even though asthmatics' cortisol levels have been found to be lower, or even normal [30-32], compared to non-asthmatics, there have also been reports of higher cortisol levels [33]. The results of these studies cannot be compared because they all focused on different aspects of the circadian pattern of cortisol secretion in relation to asthma. Stress is a factor in asthma flare-ups, according to medical professionals and researchers, for a long time. However, it is only within the last two decades that strong scientific data has emerged to support this concept. For instance, an acute negative life event (such as the loss of a close family member) raised the chance of a later asthma attack by almost 2-fold in an 18-month prospective study of children with asthma. When an acutely unfavorable incident took place amid a long period of stress, its effects were amplified. High levels of acute and chronic stress in children were associated with a 3-fold increase in the probability of an attack two weeks after the acute event [34]. Although there is new empirical evidence linking stress to the clinical characteristics of asthma, there is still much to understand about the biological mechanisms underlying this phenomenon. Previous research has thoroughly explored the role of stress in the development of asthma, which likely involves a variety of behavioral and biological mediators. Psychosocial stress is a trigger for asthma exacerbations, suggesting a potential role for HPA axis dysfunctions in stressful situations [35]. A study examined the cortisol levels of asthmatics and non-asthmatics in response to a stressful task and found that asthmatics had lower cortisol levels compared to non-asthmatics [36]. It has long been thought that the release of cortisol during stressful situations is harmful to one's health rather than beneficial. According to several theories, stress may trigger asthma because of the psychological changes that occur as a result of stress [37]. Asthma is also known to be triggered by smoking, and people often turn to smoking to relieve stress. Asthma can be brought on by panic attacks brought on by excessive stress. When cortisol is released for a long period of time, the receptor may become downregulated or less sensitive.

## CONCLUSION

We found that the incidence of hypertension and diabetes was high while the incidence of asthma was low, along with a considerable number of patients unaware of their hypertension and diabetes. Moreover, patients suffered from multiple sources of stress in their daily life affecting their disease progression. Future studies may explore the relationship between cortisol levels and the incidence of hypertension and diabetes, as well as the gender-related incidence. While our study has some limitations, such as focusing on male participants only, including a relatively small population, and being limited to individuals above 18 years of age, it is still valuable in emphasizing the importance of awareness and literacy in improving outcomes related to these health challenges.

## Personal thanks

The authors would like to express their gratitude to the University of Alkafeel and its staff members for their assistance in data collection and measurements.
